# Does Controlling for Temporal Parameters Change the Levels-of-Processing Effect in Working Memory?

**DOI:** 10.5709/acp-0182-3

**Published:** 2016-03-31

**Authors:** Vanessa M. Loaiza, Valérie Camos

**Affiliations:** 1 Department of Psychology, University of Fribourg, Switzerland; 2 Department of Psychology, University of Essex, Colchester, UK

**Keywords:** working memory, episodic memory, levels of processing

## Abstract

The distinguishability between working memory (WM) and long-term memory has been a frequent and long-lasting source of debate in the literature. One recent method of identifying the relationship between the two systems has been to consider the influence of long-term memory effects, such as the levels-of-processing (LoP) effect, in WM. However, the few studies that have examined the LoP effect in WM have shown divergent results. This study examined the LoP effect in WM by considering a theoretically meaningful methodological aspect of the LoP span task. Specifically, we fixed the presentation duration of the processing component a priori because such fixed complex span tasks have shown differences when compared to unfixed tasks in terms of recall from WM as well as the latent structure of WM. After establishing a fixed presentation rate from a pilot study, the LoP span task presented memoranda in red or blue font that were immediately followed by two processing words that matched the memoranda in terms of font color or semantic relatedness. On presentation of the processing words, participants made deep or shallow processing decisions for each of the memoranda before a cue to recall them from WM. Participants also completed delayed recall of the memoranda. Results indicated that LoP affected delayed recall, but not immediate recall from WM. These results suggest that fixing temporal parameters of the LoP span task does not moderate the null LoP effect in WM, and further indicate that WM and long-term episodic memory are dissociable on the basis of LoP effects.

## Introduction

Working memory (WM) is thought to support complex cognition by means of brief and limited maintenance and processing of information, whereas long-term episodic memory (EM) refers to explicit memory of information that is no longer actively maintained in WM. Recently, researchers have become particularly interested in the relationship between WM and EM, especially with regard to the relative distinction between the two constructs (Loaiza & McCabe, 2012; Loaiza, McCabe, Youngblood, Rose, & Myerson, 2011; McCabe, 2008; Rose & Craik, 2012; Rose, Myerson, Roediger, & Hale, 2010). One promising method of investigating this relationship has been to examine factors known to affect retrieval from EM in the context of WM paradigms. These manipulations include the levels-of-processing (LoP) effect—that is, the retrieval advantage for information that is studied with regard to its deeper, more meaningful characteristics (e.g., its semantic meaning) compared to more shallowly studied information (e.g., its visual characteristics; Craik & Tulving, 1975). Although well-replicated in the EM literature, studies concerning the LoP effect in WM have yielded divergent results (Loaiza et al., 2011; Rose, Buchsbaum, & Craik, 2014; Rose & Craik, 2012; Rose, Craik, & Buchsbaum, 2015; Rose et al., 2010), therefore leaving the status of the relationship between WM and EM still theoretically tenuous. Accordingly, this study examined whether varying a methodological element of the WM task (i.e., fixed presentation rate of the processing component) could shed light on the influence of LoP on WM.

The relationship and distinction between WM and EM is important to our understanding of the structure of declarative memory in general. While some models have emphasized a clear distinction between WM and EM (e.g., Baddeley, 2000; Barrouillet & Camos, 2015), others have considered WM to be embedded within the broader context of long-term memory (comprising semantic and episodic memory; e.g., Cowan, 1999; Oberauer, 2009), and still others see no reason to make any distinction at all (e.g., Surprenant & Neath, 2009; Ward, 2001). One recent framework posited that WM measures often require the EM-based retrieval in addition to active maintenance in WM in order for recall to be successful (Unsworth & Engle, 2007). Thus, establishing the extent to which the two constructs are distinguishable is imperative to memory research. Individual differences methods have been informative on this question (Unsworth, 2010), but recently Rose et al. ( 2010; Rose, 2013) developed the LoP span task to experimentally consider the distinction between WM and EM. The goal of the study was to investigate whether the LoP effect that is frequently observed in retrieval from EM would also be evident in WM recall. A dissociation between WM and EM on the basis of LoP would suggest that they are different. Within the LoP span task, Rose et al. (2010) were able to test WM under different LoP conditions and also to examine the consequences of this manipulation on EM by administering a delayed recognition test. Rose et al. modeled the LoP span task after typical complex span tasks that require participants to try to briefly maintain and recall memoranda while also engaging in a concurrent processing component (Conway et al., 2005). Specifically, the LoP span task presented a memorandum (*bride*) in red or blue font for 1.75 s before presenting two processing words (*dried* and *groom*) alongside one another on the screen for an unfixed amount of time. That is, the processing words remained on screen until the participant’s response. One of the processing words was in red font and the other in blue font, and both were intended to serve as the processing component in the LoP span task. Specifically, participants were asked to make a decision between the two words relative to the memorandum regarding the match between the font color (shallow level of processing), rhyme (intermediate level of processing), or semantic meaning (deep level of processing) of the words. Results indicated the typical LoP effect in EM: greater recall was exhibited as the LoP deepened, but no such effect in WM (see also Rose, 2013). Rose et al. (2010) interpreted the LoP dissociation as evidence that WM and EM are distinct.

To verify these results in more traditional span tasks, Loaiza et al. (2011) examined the LoP effect using the reading span and operation span tasks. These tasks follow the aforementioned typical procedure of requiring participants to briefly maintain and recall memoranda while engaging in a concurrent processing component. In the reading span task, participants had to read sentences (i.e., the processing component) with the last word of each sentence representing the memorandum. These sentences were either deeply or shallowly related to the memorandum (e.g., “The brother of one of your parents is an UNCLE” vs. “A word made up of five letters is UNCLE”) and were presented for an unfixed period of time. In a second experiment, an operation span task was presented that required participants to make deep or shallow judgments on the memoranda (“Is this word a living thing?” vs. “Does this word have more than one vowel?”) within 2 s while solving arithmetic problems that were presented for unfixed periods of time (i.e., the processing component). After immediately recalling the memoranda, participants completed a delayed recall test to examine the LoP effect in EM. The results indicated a LoP effect in both EM and WM. These results obviously conflicted with Rose et al. (2010), and instead suggested that both WM and EM were sensitive to the LoP of the memoranda.

Subsequent research has investigated the source of the divergent results. One factor that was examined was the differences in the relative attentional demands between the processing components used in the various WM paradigms. However, the results have not been definitive, due to divergent findings (Camos, Mora, & Loaiza, 2015; Rose & Craik, 2012; Rose et al., 2014). Thus, the literature is still presently unclear regarding the true nature of the LoP effect on WM recall, which is troubling for determining the nature of the relationship between WM and EM. The aim of the present study was to examine another methodological element as a potential culprit for the disparity in the findings. This particularly theoretically meaningful factor, namely, the temporal parameters of the task, concerns the possibility that fixing the presentation duration of the processing component in Rose and colleagues’ (2010) LoP span task may moderate the LoP effect in WM recall.

This methodological constraint in WM paradigms has had an important theoretical impact. First, fixing the presentation rate of the processing component in a complex span task paradigm has shown dramatic effects on WM capacity. That is, relative to self-paced (i.e., the participant herself advances the task) and experimenter-paced (i.e., the experimenter advances the task at the response of the participant) processing components, fixing the pace of the processing component in the computer program a priori often strongly reduces WM capacity (Barrouillet, Bernardin, & Camos, 2004; McCabe, 2010). Among the competing explanations for this effect is that fixing the temporal parameters greatly constrains participants’ ability to engage in attention-based maintenance (Barrouillet et al., 2004) and elaborative strategies (Lépine, Barrouillet, & Camos, 2005; St Clair-Thompson, 2007) to keep the memoranda active in WM. Several studies have further demonstrated that WM tasks with fixed temporal parameters are better measures of WM capacity than those with unfixed (self-paced or experimenter-paced) parameters, particularly in terms of their predictive utility for other measures of higher-order cognition (Barrouillet, Lépine, & Camos, 2008; Barrouillet, Plancher, Guida, & Camos, 2013; Lépine et al., 2005; McCabe, 2010). This is most likely because WM tasks that do not have fixed temporal parameters afford the use of strategies that confound the construct and attenuate its relation with cognitive activities. Other studies have indicated that a model with two different underlying latent factors either better accounts for the variability common to fixed-pace and unfixed-pace tasks (Bailey, 2012) or is not significantly different than a model with a single latent factor (Lucidi, Loaiza, Camos, & Barrouillet, 2014). Thus, in three different regards, the literature has shown that fixed and unfixed tasks are not identical; in fact, they often differentiate in terms of overall WM capacity (e.g., Barrouillet et al., 2004), predictive utility (e.g., McCabe, 2010), and in latent structure (e.g., Bailey, 2012). Such results highlight the possibility that complex span tasks with unfixed temporal parameters do not measure WM either as accurately as or similarly to tasks with fixed temporal parameters.

This possibility has important implications for the topic at hand: It is possible that the unfixed administration could obfuscate the LoP effect in recall from WM during the LoP span task. That is, if fixing the temporal parameters of complex span tasks provides a more valid measure of WM capacity (Conway et al., 2005), then fixing the temporal parameters of the LoP span task could increase the likelihood that the LoP effect is observed in WM. This could occur for several reasons. As mentioned previously, varying the temporal parameters of the processing component tends to vary the likelihood of strategy use during complex span tasks (Friedman & Miyake, 2004; St Clair-Thompson, 2007) and in turn reduces the validity of the WM measure (Lépine et al., 2005; McCabe, 2010; St Clair-Thompson, 2007). Accordingly, unfixed temporal parameters during the LoP span task could unintentionally introduce the possibility that the participants use strategies that interfere with the true LoP effect. For example, increasing the ability to rehearse information from the primary task of remembering the memoranda, either by instruction (Turley-Ames & Whitfield, 2003) or by self-pacing the task (Barrouillet et al., 2004; Engle, Cantor, & Carullo, 1992), tends to increase WM capacity. However, it is also known that rehearsal is a shallow, phonological method of maintenance in WM (e.g., Baddeley, 1966; Camos, Mora, & Oberauer, 2011) that, if allowed to occur in an unfixed setting, could diminish the influence of semantic processing in the LoP span task. Indeed, in their recent studies, Rose and colleagues (2014, 2015) showed that instructions to rehearse the word during an interval between the LoP judgment and recall of the word yielded a null LoP effect. Moreover, such strategy use could even change the validity of the WM measure (e.g., Bailey, 2012; Engle et al., 1992; Friedman & Miyake, 2004) to the extent that it may no longer properly reflect the construct. Thus, from a broader theoretical perspective, the validity of the WM measure must be ensured in order to clarify the effect of LoP in WM.

More recent studies concerning LoP in WM have fixed the presentation rate of their processing components (Camos et al., 2015; Rose et al., 2014, 2015), sometimes even with very short presentation rates. Just as in the Loaiza et al.’s (2011) study using complex span tasks, memoranda in these studies were judged according to deep or shallow processing conditions and followed by an unrelated processing component. However, unlike the previous LoP studies that did not fix the timing of the processing component (Loaiza et al., 2011; Rose et al., 2010), the processing component was fixed at 4 s (Camos et al., 2015) or 10 s (Rose et al., 2014, 2015) between presentation of the memoranda and/or immediate recall attempt. The results of these studies that fixed the temporal parameters of the complex span task typically observed a LoP effect in WM. However, the tasks in these studies were more traditional complex span tasks in which the processing component usually distracts attention away from the memoranda, whereas the processing component in the LoP span task specifically refers back to the memoranda. Accordingly, it may be possible that the issue of disparity in the results regarding the LoP effect in WM could be simply addressed by fixing the time allotted for the processing decisions. If the LoP effect emerged in such a scenario, then it would suggest that fixing the presentation rate of the processing component is an important factor for the LoP effect in WM. That is, even at very short intervals of a processing component (Camos et al., 2015), a LoP effect in WM could be observed as long as the processing component is fixed in order to eliminate any other processes or strategies that are irrelevant to the LoP engendered by the task. However, if Rose et al.’s (2010) results are replicated such that no LoP effect appears in WM recall, then it would also provide the field with more data regarding the robustness of the originally reported results even when fixing the temporal parameters of the task. Accordingly, our study aimed to replicate the design of Rose and colleagues’ (2010) study except for one crucial methodological, but theoretically meaningful, difference: a fixed presentation of the processing component during which participants make LoP judgments. We sought to clarify that the results reported thus far persist even when accounting for theoretically meaningful methodological parameters that have been shown to, at the very least, reduce WM capacity (e.g., Barrouillet et al., 2004) and, at most, yield two different latent variables suggesting overlapping but still independent measures of WM capacity (Bailey, 2012). This would allow researchers to better consider their theoretical implications, especially for as important a topic as the distinction between WM and EM. Thus, in the present experiment, we examined whether fixing the presentation rate of the processing component decisions in the LoP span task would yield a LoP effect in WM. This presentation rate and the characteristics of the memoranda used in the experiment were ascertained in two pilot experiments (see Appendix).

## Method

The experiment presented a LoP span task similar to Rose et al. (2010), except that the presentation of the processing component was fixed for the respective LoP decisions (i.e., visual and semantic). Thus, we were able to test whether the null LoP effect in WM is changed when fixing the presentation rate of the processing component.

### Participants and Design

Twenty-nine students at the Université de Fribourg (three men, *M*_age_ = 20.86, *SD* = 1.65) received partial course credit for participating. One participant was excluded due to technical problems during the experiment. None of the participants had participated in the pilot studies.

The two independent variables were (1) the time of test (immediate or delayed recall of the memoranda), and (2) the LoP (shallow or deep processing, i.e., decisions concerning visual or semantic relations between the memoranda and the processing words, respectively), and were manipulated within-subjects. The dependent variable of major interest was recall of the memoranda. We additionally measured response times (RTs) and accuracy for the LoP judgments. Accuracy on the LoP judgments was reduced when participants made an incorrect judgment or when their responses were too slow (i.e., a “time out”).

### Materials and Procedure

The memoranda for the experiment were developed to closely resemble those of Rose et al. (2010; see Appendix). One-hundred twenty critical experimental words (60 target memoranda, 60 semantic associates) were randomly assigned and fixed to one of two blocks that were counterbalanced for order of presentation and processing condition across participants. The words between the blocks were statistically equivalent in terms of forward associative strength, length and syllables of the targets and associates, word frequency, and concreteness [except for the associate syllables, *t*(58) = 2.11, *d* = 0.18, *p* = .04]. The non-semantic alternative was randomly assigned and pre-fixed to a target-associate word pair. The target memoranda and their semantic and non-semantic associates were pseudo-randomly arranged into trials and randomly presented within the blocks. Each block had six trials of five memoranda, with two practice trials preceding each block.

The procedure was modeled after the original LoP span task (Rose et al., 2010). A to-be-remembered target word (e.g., *bottle*) was presented for 1 s and read aloud. After a 250 ms ISI, two processing words (e.g., *wine* and *work*) appeared on either side of the screen in red or blue font. The font color of the target and processing words and the position of the processing words on the left- or right-hand side of the screen were randomly assigned. Depending on the condition, participants were asked to choose the word that either matched the previous target’s color (shallow condition) or that was semantically more related to the target (deep condition). Participants made their processing decision using a marked left- or right-hand key corresponding to the position of the word on screen. They were instructed to make their decision as quickly and accurately as possible, with the processing words presented for 620 ms or 970 ms in the shallow and deep conditions, respectively. This timing of the processing words was derived from the pilot study in which participants completed the deep and shallow processing component without a memory load (see Appendix). A fixed 750 ms inter-stimulus interval (ISI) separated the presentation of the processing words and the next target word. After five targets were presented, participants were prompted to recall the target words from the trial in their order of presentation by typing them into the computer using the keyboard.

A 2-min distracter followed each block, wherein participants solved simple arithmetic problems (e.g., 43 + 10 =?) and typed their responses using the keyboard. Afterward, participants were instructed to freely recall as many of the target words from the preceding block as possible, with no regard to the order of the words.

## Results

In order to compare between immediate and delayed recall, we scored immediate recall without regard to serial order (McCabe, 2008). However, it should be noted that the serial recall pattern was similar to the pattern reported here. All reported significant results met a criterion of *p* < .05.

**Figure 1.**
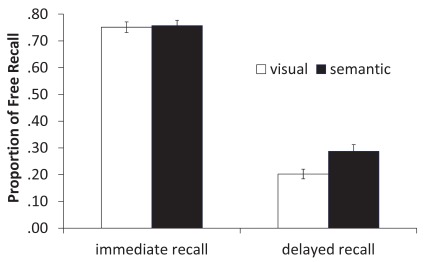
Proportion of free recall from visual and semantic conditions during immediate and delayed recall. Error bars reflect one standard error of the mean.

As in the second pilot experiment (see Appendix), participants were significantly faster to respond to the processing words in the visual (453 ms, *SD* = 72) than semantic (765 ms, *SD* = 77) conditions, *t*(27) = −25.15, *d* = 4.18, although there was no difference in response accuracy (79%, *SD* = 20% vs. 79%, *SD* = 15%, respectively), *t* < 1.00. In order to assess recall performance, we ran a 2 (time: immediate, delayed) × 2 (LoP: visual, semantic) repeated-measures analysis of variance. Immediate recall was significantly greater than delayed recall, *F*(1, 27) = 572.52, η_p_² = .96, and recall was greater overall in the semantic than visual conditions, *F*(1, 27) = 8.27, η_p_² = .24. Critically, the two-way interaction was significant, *F*(1, 27) = 7.69, η_p_² = .22, such that the typical LoP effect occurred in delayed recall, *F*(1, 27) = 11.22, η_p_² = .29, but not in immediate recall, *F* < 1 (see Figure 1). Thus, we replicated the results of the original LoP span task.

## Discussion

The critical result of the reported study was that the null LoP effect in WM did not change when fixing the presentation rate of the shallow and deep processing decisions during the LoP span task. This does not only replicate Rose et al.’s (2010) finding, but it also shows that their pattern of results is robust under several different conditions: Most notably, under fixed temporal parameters, but also using French stimuli and a delayed free recall test rather than a recognition test as in Rose et al. (2010). In sum, the findings suggest that fixing the presentation rate does not explain the absence of the LoP effect in WM when tested in the LoP span task.

Given the importance of fixed temporal parameters in the literature with regard to not only the effect on WM capacity (Barrouillet et al., 2004), but also the predictive utility of the WM measures (McCabe, 2010) and their latent structure (Bailey, 2012), this finding is striking. It suggests that the original distinction of LoP on WM versus EM still persists even when the opportunity to engage in other strategies that could have diminished the effect, such as rehearsal, is more strictly controlled. Moreover, the replicated results also indicate that the LoP span task may be somewhat unique among traditional WM span tasks. Indeed, when fixing the temporal parameters of the interval following the LoP judgment and before recall to a retention interval of 10 s, Rose and colleagues (2014, 2015) showed that the LoP effect in WM recall of one word increased with an increasing attentional demand imposed by the processing component during this interval. However, there was no LoP effect when the participants were instructed to rehearse the word during this interval. Camos and colleagues (2015) required participants to make LoP decisions on five words that were interspersed by processing components that also varied in their attentional demand but were of fixed duration (4 s). Like in Rose and colleagues (2014, 2015), a LoP effect in WM was observed, although the effect did not vary with the attentional demand of the processing component. Thus, these studies show that fixing temporal parameters can yield a LoP effect in WM. The only case that the LoP effect in WM was not observed was when the participants were instructed to rehearse the memoranda (Rose et al., 2014, 2015).

What, then, may explain the difference between the divergent results concerning the LoP effect? The LoP span task and the traditional WM span tasks differ in one fundamental characteristic. In the LoP task, participants have to refer back to the memorandum to make a correct processing decision. Thus, the processing component in the LoP span task sustains the active maintenance of the memoranda, whereas the processing component in complex span tasks more typically distracts attention away from actively maintaining the memoranda. As previously discussed, Rose and Craik (2012) argued that the LoP effect depends on active maintenance in WM: The LoP effect should emerge in WM when active maintenance processes are diminished, thereby, requiring the participants’ reliance on EM resources and the corresponding effects that are already well-established in the EM literature, such as the LoP effect. However, the processing component in the LoP span task does not involve distraction but instead prompts sustained maintenance of the last-presented word in order to make a LoP decision. Rose and colleagues (2014, 2015) have already demonstrated that being able to actively maintain the memoranda in WM, such as via rehearsal, does not yield a LoP effect. Likewise, the nature of the LoP span task paradigm of sustained maintenance rather than distraction during the processing component makes it more likely that a LoP effect could not be demonstrated in this task. Indeed, the present results add to the existing literature that has demonstrated that the null LoP effect in the LoP span task does not change even with increased number of memoranda per trial or when the LoP decisions are more comparable in duration to one another (Rose et al., 2010). The null LoP effect in WM is also not affected by individual differences in WM capacity (Rose, 2013). Thus, it is possible that the conditions of the WM paradigms used, such as the LoP span task, can determine whether the LoP effect is evident in WM tasks.

Far from being merely a task-specific factor, however, this possibility is theoretically meaningful: Congruent with Rose and colleagues’ predictions, uninterrupted active maintenance of memoranda yields a null LoP effect in WM tasks. That is, directing attention back to the memoranda to make a successful LoP decision involves consistent maintenance of the memoranda that is never disrupted, and consequently no LoP effect is observed. Conversely, when active maintenance is disrupted (e.g., by an unrelated task), the LoP effect emerges (Camos et al., 2015; Loaiza et al., 2011; Rose et al., 2014, 2015). This suggests that EM-based factors such as LoP are only influential on WM task performance if information is no longer actively maintained. The findings of the current study are therefore congruent with the suggestion that there is less of a distinction between WM and EM tasks if active maintenance in WM is disrupted because that information must be refreshed or reactivated in WM after having been disrupted, thereby requiring EM resources. Moreover, the current study extends this notion through the finding that the null effect persists even when controlling for auxiliary strategies that are unimportant to and sometimes change the nature of the WM measure via fixed temporal parameters. Thus, the results are important because they indicate that the null LoP effect in the LoP span task paradigm is robust, and is theoretically relevant given the more recent literature about active maintenance in WM. That is, the results support the notion that WM and EM are distinguishable to the extent that active maintenance in WM is never disrupted. WM and EM are less distinguishable, however, when maintenance is disrupted even through the simplest of concurrent activities (Camos et al., 2015), and in turn yields a LoP effect in WM tasks that is similarly present in EM tasks.

In summary, the present study indicates that the null LoP effect in WM tasks persists even when fixing the presentation rate of the processing component in the LoP span task, and this is consistent with recent evidence that never disrupting active maintenance in WM yields a null LoP effect (Rose et al., 2014). This finding underlines the importance of active maintenance as a factor that distinguishes WM and EM: If active maintenance in WM is never disrupted, EM-based processes (such as those important for the LoP effect) do not affect WM performance. A remaining issue for further investigation is whether the LoP effect in WM tasks is moderated when varying the use of active maintenance mechanisms in WM (Camos et al., 2015; Rose et al., 2014, 2015). However, this study provides insights about the LoP effect within the context of the LoP span task, and further demonstrates the replicability of the pattern even given when changing a crucial methodological element of the task.

## Appendix

This Appendix comprises the two pilot studies that were conducted in order to establish the characteristics of the memoranda and the presentation rates used in the main experiment. The first pilot study assessed concreteness of the words to ensure compatibility with Rose et al. (2010) and established concreteness ratings of French words (Bonin et al., 2003). The second pilot study determined the presentation rate for the main experiment.

Method

Participants

Twenty-two students completed concreteness ratings, while another sample of 19 students completed the processing component of the LoP span task to assess response speed and accuracy. All participants were 18-30 years old, and were native French speakers. Participants received partial course credit for their participation. Participants were not allowed to complete both pilot studies. Of the second sample, two participants were excluded due to mean RTs or accuracy exceeding 3 standard deviations from the group mean on one of the processing conditions.

Materials and Procedure

After first completing a separate study, the first sample of participants received a list of 233 French words and rated them regarding their concreteness on a five-point Likert scale (1 = *not very concrete*, 5 = *very concrete*). The words were pre-selected according to their forward associative strength to at least one other word in the sample, ranging between 30-60% (Ferrand, 2001; Ferrand & Alario, 1998; Ferrand et al., 2010), in order to later compose the processing words for the LoP span task. Of these 233 words, 124 were represented in Bonin and colleagues’ (2003) established concreteness ratings of French words, whereas 109 were not, and thus it was also important to determine a high correspondence between the pilot study and the established ratings. The words were presented in a fixed randomized order as a paper-and-pencil test.

The second sample of participants also completed a separate study not discussed here before beginning the pilot study. Sixty trials were divided between two blocks that presented the deep or shallow processing conditions. The order of the processing conditions and the words were counterbalanced across participants, and the presentation of the target and its processing words was random within each block. Before each block, five practice trials were shown. During each block, a word (*bouteille* or *bottle* in English) was presented at the center of the screen for 1 s in red or blue font. After a 250 ms interstimulus interval (ISI), two processing words were simultaneously shown on either side of the screen, with one word in red font and one word in blue font. The font colors were randomly assigned to the words. In addition, one of the processing words was related to the previous target (*vin* or *wine* in English) while the other was not (*travail* or *work* in English). The participants were asked to press the left or right key on a keyboard, corresponding to the position of the relevant processing word on the screen. In the shallow condition, participants selected the processing word that was of the same color as the preceding target word, while in the deep condition participants selected the processing word that was semantically related to the preceding word. Although the task was self-paced, in order to establish the presentation rates of the main study, participants were instructed to respond as quickly and accurately as possible. All of the words were between 3-10 letters and 1-2 syllables in length.

Results and Discussion

The first pilot study showed a high correspondence between our sample’s average concreteness ratings (*M* = 4.84, *SD* = 0.37) and the norms of Bonin and colleagues [2003; *M* = 4.66, *SD* = 0.36), *r*(124) = .82, *p* < .01]. Of the 233 total words, 120 critical experimental words were selected for use in the main experiment (60 target memoranda, 60 processing words) according to their high concreteness ratings (*M* = 4.69, *SD* = 0.52) and their moderate forward associative strength between the target and semantic processing word (*M* = 41.71, *SD* = 8.55). Thus, the experimental words were similar to those used in Rose et al. (2010).

As expected, the second pilot study showed that participants responded more quickly during visual (440 ms, *SD* = 177) than semantic (784 ms, *SD* = 185) conditions, *t*(16) = -6.50, *d* = 1.90, *p* < .01. However, participants were only numerically more accurate in visual (97%, *SD* = 5%) than semantic (94%, *SD* = 6%) conditions, *t*(16) = 1.79, *d* = 0.40, *p* = .09. Thus, the presentation rate for the processing decision in the main experiment was defined using the mean RT plus one standard deviation for each condition: 620 ms and 970 ms for visual and semantic conditions, respectively.
